# 4-Hydroxynonenal Contributes to Angiogenesis through a Redox-Dependent Sphingolipid Pathway: Prevention by Hydralazine Derivatives

**DOI:** 10.1155/2017/9172741

**Published:** 2017-04-05

**Authors:** Caroline Camaré, Corinne Vanucci-Bacqué, Nathalie Augé, Mélanie Pucelle, Corinne Bernis, Audrey Swiader, Michel Baltas, Florence Bedos-Belval, Robert Salvayre, Anne Nègre-Salvayre

**Affiliations:** ^1^INSERM I2MC, UMR-1048, Toulouse, France; ^2^Université Paul Sabatier Toulouse III, Toulouse, France; ^3^CNRS UMR 5068, Laboratoire de Synthèse et Physico-Chimie de Molécules d'Intérêt Biologique, Toulouse, France

## Abstract

The neovascularization of atherosclerotic lesions is involved in plaque development and may contribute to intraplaque hemorrhage and plaque fragilization and rupture. Among the various proangiogenic agents involved in the neovascularization process, proatherogenic oxidized LDLs (oxLDLs) contribute to the formation of tubes* via* the generation of sphingosine 1-phosphate (S1P), a major mitogenic and proangiogenic sphingolipid mediator. In this study, we investigated whether 4-hydroxynonenal (4-HNE), an aldehydic lipid oxidation product abundantly present in oxLDLs, contributes to their proangiogenic properties. Immunofluorescence analysis of human atherosclerotic lesions from carotid endarterectomy showed the colocalization of HNE-adducts with CD31, a marker of endothelial cells, suggesting a close relationship between 4-HNE and neovessel formation. In vitro, low 4-HNE concentration (0.5–1 *µ*M) elicited the formation of tubes by human microvascular endothelial cells (HMEC-1), whereas higher concentrations were not angiogenic. The formation of tubes by 4-HNE involved the generation of reactive oxygen species and the activation of the sphingolipid pathway, namely, the neutral type 2 sphingomyelinase and sphingosine kinase-1 (nSMase2/SK-1) pathway, indicating a role for S1P in the angiogenic signaling of 4-HNE. Carbonyl scavengers hydralazine and bisvanillyl-hydralazone inhibited the nSMase2/SK1 pathway activation and the formation of tubes on Matrigel® evoked by 4-HNE. Altogether, these results emphasize the role of 4-HNE in the angiogenic effect of oxLDLs and point out the potential interest of pharmacological carbonyl scavengers to prevent the neovascularization process.

## 1. Introduction

Angiogenesis, that is, the formation of new capillaries from preexisting blood vessels, is required for embryonic vascular development and wound healing and is involved in the pathophysiology of various diseases, such as diabetic retinopathy, cancer, and atherosclerosis [1]. In human normal arteries, the adventitial vasa vasorum constitute a microvascular network that supplies oxygen and nutrients to the outer part of the arterial wall. In contrast, the inner part of the arterial wall does not contain (or only few) capillaries and is fed by diffusion from the lumen [2]. Angiogenesis from vasa vasorum may be induced by an increased thickness of the vascular wall. In atherosclerotic lesions, the relative local hypoxia, which results from insufficient oxygen and nutrient diffusion and from the enhanced demand due to increased metabolism of inflammatory cells, activates the HIF/VEGF pathway and the subsequent angiogenic response [3–7]. Interestingly, in atherosclerosis prone areas of coronary arteries, hypercholesterolemia induces neovascularization in the very early steps of intima hyperplasia, before the thickening of the vascular wall [8, 9]. This suggests that, beside the activation of hypoxia-inducible transcription factors that enhance the expression of angiogenic factors [10, 11], some stimuli associated with hypercholesterolemia during early steps of atherosclerosis may induce an angiogenic signaling.

Atherosclerosis is a long and complex multifactorial process which involves several classical pathogenic events, including endothelial activation and injury, leukocyte recruitment and activation, oxidative stress, LDL oxidation and modification, macrophagic foam cell formation, local inflammation, smooth muscle cell migration and proliferation, and extracellular matrix (ECM) synthesis [12–16]. Early atherosclerotic lesions are characterized by clusters of lipid-laden macrophagic cells that form the fatty streaks, whereas advanced atherosclerotic plaques are constituted by a central core containing extracellular lipids (mainly cholesterol) and cell debris, surrounded by macrophagic cells and a collagenous fibrous cap [17, 18].

In atherosclerotic prone areas, activated vascular and inflammatory cells induce a local oxidative stress and LDL oxidation. In vitro, oxidized lipids exhibit a variety of biological properties, suggesting their potential role in the progression of atherosclerotic lesions [14, 19–22]. Various oxidized lipids are generated during the peroxidation of polyunsaturated fatty acids, in particular the unsaturated aldehyde 4-hydroxynonenal (4-HNE), which is highly reactive with thiol and amino groups and forms adducts with proteins and other cellular components [23–25]. 4-HNE-adducts are abundant in the center of the plaque and in macrophagic cells of human carotid atherosclerotic lesions [26, 27].

As atherosclerotic neovascularization develops mainly in lipid-rich atheromatous and inflammatory areas, this suggests that the association of atheromatous lipids with local inflammation may play a role in angiogenesis. Oxidized phospholipids exhibit proinflammatory [28] and proangiogenic properties [29]. Oxidation derivatives of arachidonic acid may act as initiators of atherogenesis and trigger endothelial cell proliferation and capillary network formation [30]. Oxidized LDLs (oxLDLs) exhibit a dual dose-dependent angiogenic effect, since low oxLDL concentrations are angiogenic, while higher concentrations are not angiogenic and are rather cytotoxic [31–35]. The angiogenic effect of oxLDLs is mediated by their binding to LOX-1 that triggers the activation of signaling pathways involving NAD(P)H oxidase, p38-MPAK, PI3 kinase/Akt/eNOS, and neutral sphingomyelinase-2/sphingosine kinase-1/sphingosine-1-phosphate (nSMase2/SK1/S1P) [31, 33, 35, 36] and the expression of angiogenic genes (e.g., VEGFR-2, PDGFR, NOTCH-1, and NRP-1) [29, 34].

Low concentration of 4-HNE upregulates VEGF expression and thus potentially induces angiogenesis, in retinal pigment epithelial cells [37]. In contrast, higher 4-HNE concentration upregulates chondromodulin-1 and inhibits angiogenesis [38].

We aimed to evaluate whether 4-HNE exerts a pro- or antiangiogenic effect on cultured endothelial cells, to investigate the 4-HNE-induced angiogenic signaling and to prevent the angiogenic effect by carbonyl scavengers and signaling inhibitors.

## 2. Methods

### 2.1. Chemicals

Matrigel was from BD Biosciences (Le-Pont-de-Claix, France). Calcein-AM bioreagent, trolox, diphenylene iodonium (DPI), GW4869, Vas2870, and hydralazine were from Sigma, and 4-HNE was from Calbiochem, [methyl-^14^C]choline-sphingomyelin was from Perkin-Elmer. 5- (and 6-)Carboxy-2′,7′-dichlorodihydrofluorescein diacetate (H2DCFDA), was from Molecular Probes (Invitrogen France). The anti-CD31 was from Abcam, the anti-4-HNE Michael adducts were from Calbiochem, the anti-LOX-1 antibody (aLox1 Ab) was from R&D Systems, and the anti-CD68 was from Thermo Fisher Scientific. Alexa Fluor 488-conjugated and Alexa Fluor 546-conjugated secondary antibodies were from Invitrogen. Cell culture reagents and other materials were from WWR or Sigma. Bisvanillin (BV) and bisvanillyl-hydralazone (BVH) were synthesized as reported [39].

### 2.2. Cell Culture

Human microvascular endothelial cells (HMEC-1) (CDC, Dr. Candal, Atlanta, US) were grown in MCDB131 culture medium supplemented with 10% heat inactivated fetal bovine serum (FBS), 100 U/ml penicillin, and 100 *µ*g/ml streptomycin.

### 2.3. LDL Isolation and Oxidation Parameters

LDLs were prepared by ultracentrifugation of pool of human sera and mildly oxidized by UV irradiation [35, 36]. The extent of LDL oxidation was monitored by the determination of the thiobarbituric reactive substance (TBARS) content, using the fluorimetric procedure of Yagi [40]. The 4-HNE-adduct content was determined by ELISA (OxiSelect™ HNE Adduct Competitive ELISA Kit, Cell Biolabs Inc.), in the conditions of the manufacturer.

Under standard conditions, these oxLDLs contained 78–97 nmol lipid hydroperoxide/mg apoB, 10–15 nmol 4-HNE/mg apoB, and 8.7 nmol TBARS/mg apoB.

### 2.4. Intracellular ROS Determination

Intracellular ROS generated in cells upon treatment by oxLDL or 4-HNE were evaluated by measuring the oxidation of H_2_DCFDA-AM [35, 36]. 30 min before the end of the experiment, the probe was added to the culture medium (5 *µ*M final concentration) of HMEC-1 previously seeded on 96-well microplates. Cells were carefully washed twice with phosphate buffered saline (PBS), then 0.2 ml fresh PBS was added to each well, and the fluorescence of CFDA was measured using a fluorescence microplate reader TECAN® (excitation/emission 495/525 nm). The data are expressed as ratio of fluorescence/fluorescence of the unstimulated control.

### 2.5. Determination of nSMase2 and SK1 Activities

The activity of nSMase2 was measured using radiolabeled [methyl-^14^C]choline-sphingomyelin (Perkin-Elmer), and SK1 activity was determined in HMEC-1 lysed in ice-cold lysis buffer, after incubation with [^33^P]ATP (Perkin-Elmer), as reported [35, 36]. The [^33^P]-labeled-S1P was extracted, isolated by TLC, and counted by liquid scintillation.

Protein concentration was determined using the Bradford reagent (Bio-Rad).

### 2.6. Capillary Tube Formation

HMEC-1 were seeded (30,000 cells/well) in MCDB131 supplemented with 0.1% FCS (negative control) on 24-well plates coated with Matrigel and incubated with 4-HNE freely added to the culture medium at the indicated concentrations and the pharmacological agents, when indicated. After 24 h at 37°C, the cells were labeled with Calcein-AM (1 *µ*M, 30 minutes) and observed by fluorescence microscopy (exc. 496/em. 516, resp.), under the previously used conditions [35]. The number of capillary tubes (linked cells) was counted and reported to the total cell number.

### 2.7. Immunofluorescence and Immunohistochemistry

Serial 3 *µ*m thin sections of human advanced carotid plaques obtained after endarterectomy (Cardiovascular Surgery Department, CHU Toulouse) were characterized by hemalun/eosin staining and were incubated with the antibodies, anti-CD31, anti-4-HNE Michael adducts, and anti-CD68, and revealed using either avidin-biotin horseradish peroxidase visualization system (Vectastain, ABC Kit Elite, Vector Laboratories) or Alexa Fluor 488-conjugated and Alexa Fluor 546-conjugated secondary antibodies and confocal microscopy (Zeiss 780).

### 2.8. Statistical Analyses

The results are presented as mean ± SEM of *n* experiments. Statistical significance was estimated by analysis of variance (SigmaStat 3.5, Systat Software). When test for normality and equal variance (Kolmogorov–Smirnov) was passed, differences between means values were evaluated byone-way ANOVA (several experimental groups) followed by multiple comparisons by the Holm–Sidak test. Values of *P* < 0.05 were considered significant.

## 3. Results

### 3.1. 4-HNE and Neovascularization in Human Atherosclerotic Plaques

**Figure 1:**
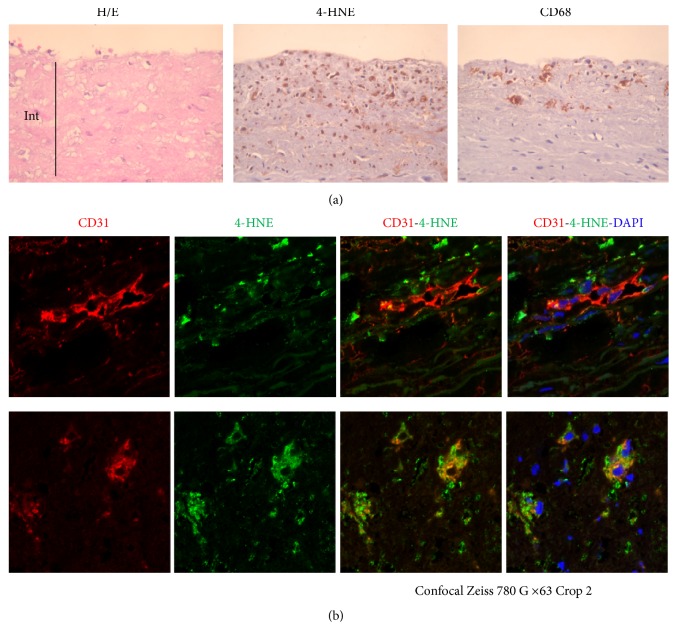
*4-HNE is colocalized with CD31 in human atherosclerotic lesions*. Paraffin sections of human carotid plaques from endarterectomy were analyzed. In (a), hematoxylin/eosin (H/E) staining and immunostaining for 4-HNE-adduct (HNE) and CD68 expression. In (b), immunofluorescence analysis of 4-HNE-adduct expression (green) and CD31 (red), with nuclei counterstaining by DAPI. Int: intima. These pictures are representative of analysis for 3 separate advanced carotid plaques.

Immunohistological studies of human atherosclerotic lesions of carotid endarterectomy show a staining for 4-HNE-adducts localized in areas labeled for CD68 (Figure 1(a)), thus suggesting that 4-HNE is generated in inflammatory areas. Confocal immunofluorescence shows that CD31-positive tubular capillary structures are surrounded by areas stained for 4-HNE-adducts (Figure 1(b), upper panels). Sometimes, 4-HNE is colocalized with CD31 (Figure 1(b), lower panels), thus suggesting that a relationship may exist between 4-HNE and angiogenesis. This led us to investigate whether 4-HNE exhibits an angiogenic effect in a model system of HMEC-1 grown on Matrigel.

### 3.2. Pro- and Antiangiogenic Effect of 4-HNE on HMEC-1 Grown on Matrigel

We used the HMEC-1 cell line in angiogenesis experiments, because these endothelial cells of microvascular origin are immortalized and stable over time, in contrast to primary endothelial cells (e.g., HUVEC), which originate from multiple donors and exhibit phenotypic changes and limited lifespan.

**Figure 2:**
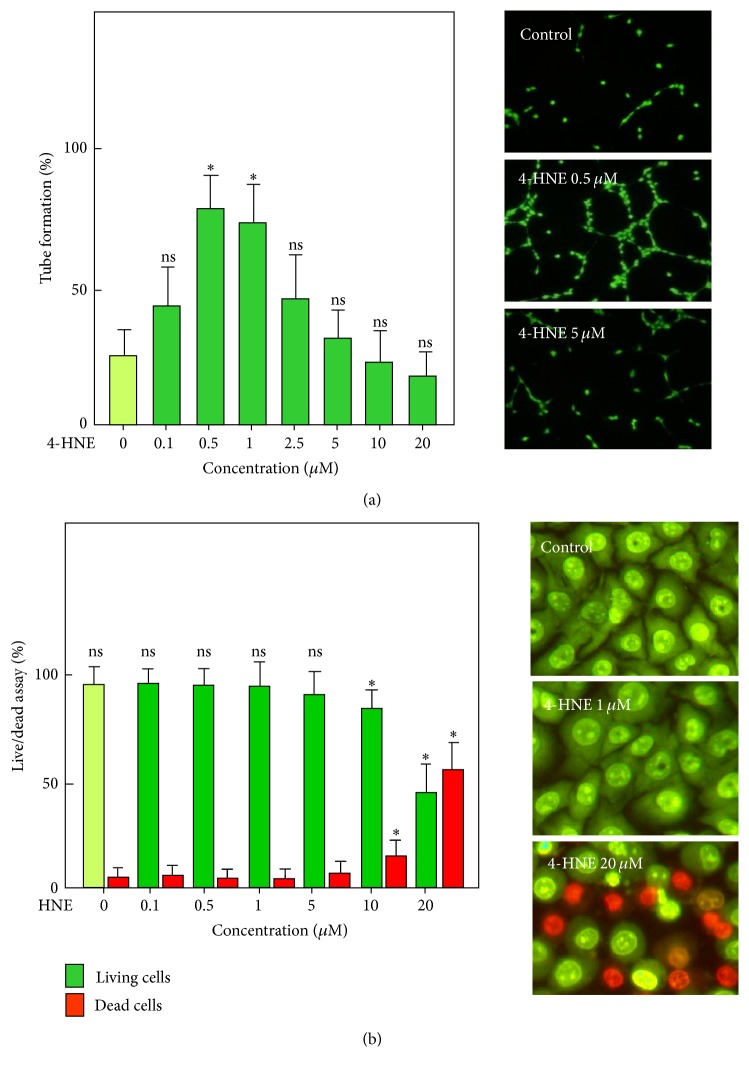
*Dual effect of 4-HNE on neocapillary formation by HMEC-1*. (a) Dose-response effect of 4-HNE on capillary tubes formed by HMEC-1. Cells were grown on Matrigel in MCDB131 culture medium supplemented with 0.1% FBS and PBS (negative control) or 4-HNE (in PBS) varying from 0.1 to 20 *µ*M. After 18 h incubation, the cells were stained with calcein (1 *μ*mol/1, 30 min) and photographed (Nikon Coolpix 995 camera) under a fluorescence microscope. Tube formation was expressed as linked cells per 100 cells. Results are means ± SEM of 6 to 8 experiments. Right panel, representative pictures of the experiments. ^*∗*^*P* < 0.05; ns: not significant. (b) Live-dead experiment on HMEC-1 stimulated by increasing 4-HNE concentrations and performed using the fluorescent DNA probes, permeant green Syto13 (0.6 *µ*M) and nonpermeant red propidium iodide (1 *µ*M). The results are expressed as the number (%) of living, apoptotic, or necrotic cells versus total cells. Right panels, representative pictures of fluorescence microscopy of HMEC-1, incubated for 18 h without (control) or with 4-HNE 1 *µ*M or 20 *µ*M. Means ± SEM of 3 experiments. ^*∗*^*P* < 0.05; ns: not significant.

4-HNE exhibited a biphasic dose-dependent effect on tube formation by HMEC-1 cells grown on Matrigel (Figure 2(a)). Under the conditions used in Figure 2, the highest angiogenic effect was observed at low concentration, between 0.5 and 1 *µ*M of 4-HNE. At concentrations higher than 1 *µ*M, the angiogenic effect decreased and was below the baseline at 10 *µ*M. The toxic effect evaluated by morphological apoptosis was detected at 10 *µ*M and higher concentrations (Figure 2(b)).

### 3.3. Intracellular ROS Mediate 4-HNE-Induced Tube Formation by HMEC-1 on Matrigel

**Figure 3:**
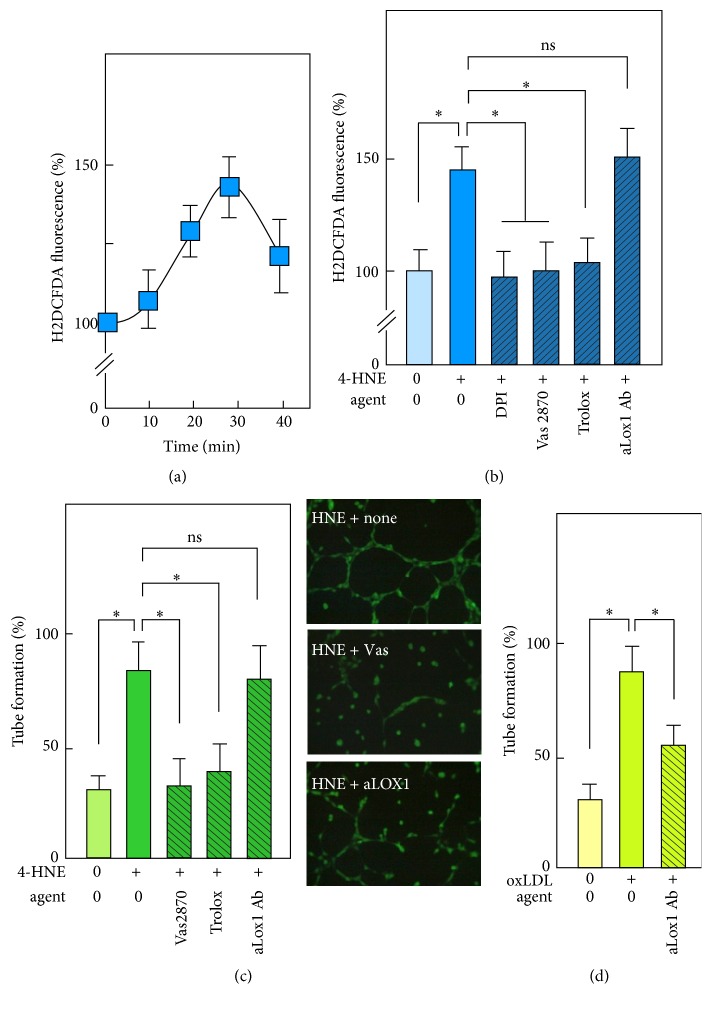
*Implication of ROS in tube formation by 4-HNE*. (a) Time-course of intracellular ROS production evoked by 4-HNE (0.5 *µ*M) in HMEC-1 and measured fluorometrically using the H2DCFDA probe (5 *µ*M final concentration). Results are expressed as % of the unstimulated control. (b) Effect of the antioxidant trolox (10 *µ*M) and of NADPH oxidase inhibitors DPI and Vas2870 (10 *µ*M each) and of the anti-Lox-1 antibody (5 *µ*g/ml) on ROS generated by HMEC-1 after 30 min of contact with 4-HNE (0.5 *µ*M). (c) Effect of trolox, DPI, and Vas2870 and anti-Lox-1 antibody, on tube formation elicited by 4-HNE (0.5 *µ*M). Representative pictures of tube formation in the presence of 4-HNE (0.5 *µ*M) and without (none) or with inhibitors Vas2870 (Vas) or anti-Lox-1 Ab (aLox1). (d) Effect of the anti-Lox-1 Ab on tube formation elicited by oxLDL (20 *µ*g/ml). Note that the anti-Lox-1 Ab has no effect on tubes formed by 4-HNE-stimulated HMEC-1 but inhibits tubes formed by oxLDL-stimulated cells. These data are means ± SEM of 5 separate experiments. ^*∗*^*P* < 0.05; ns: not significant.

4-HNE is one of the major RCCs present in oxLDLs that also exhibit angiogenic properties at low concentration [31, 35, 36]. This led us to investigate whether the same angiogenic signaling pathways were involved in 4-HNE tube formation. Low concentration of 4-HNE triggered a rise of intracellular ROS that peaked 30 min after 4-HNE addition to the culture medium (Figure 3(a)). 4-HNE-induced ROS were involved in the angiogenic response, as shown by the inhibitory effect of the cell-permeant antioxidant Trolox and the NADPH oxidase inhibitors DPI and Vas2870 that blocked both ROS generation and tube formation (Figures 3(b) and 3(c)). The inhibition of intracellular ROS and tube formation by DPI and Vas2870 suggest that ROS are generated by a NADPH oxidase, like those triggered by oxLDLs, but through a different mechanism. It may be noted that low oxLDL concentration triggers similar signaling and angiogenic effect through a LOX-1-dependent mechanism [31, 35], but, under the experimental conditions used here, 4-HNE-induced ROS signaling and tube formation were not inhibited by anti-LOX-1 antibody (Figures 3(b) and 3(c)), while oxLDL-induced capillary tube was inhibited by anti-LOX-1 antibody (Figure 3(d)).

### 3.4. 4-HNE Activates the Neutral Sphingomyelinase-2/Sphingosine Kinase-1 Pathway

**Figure 4:**
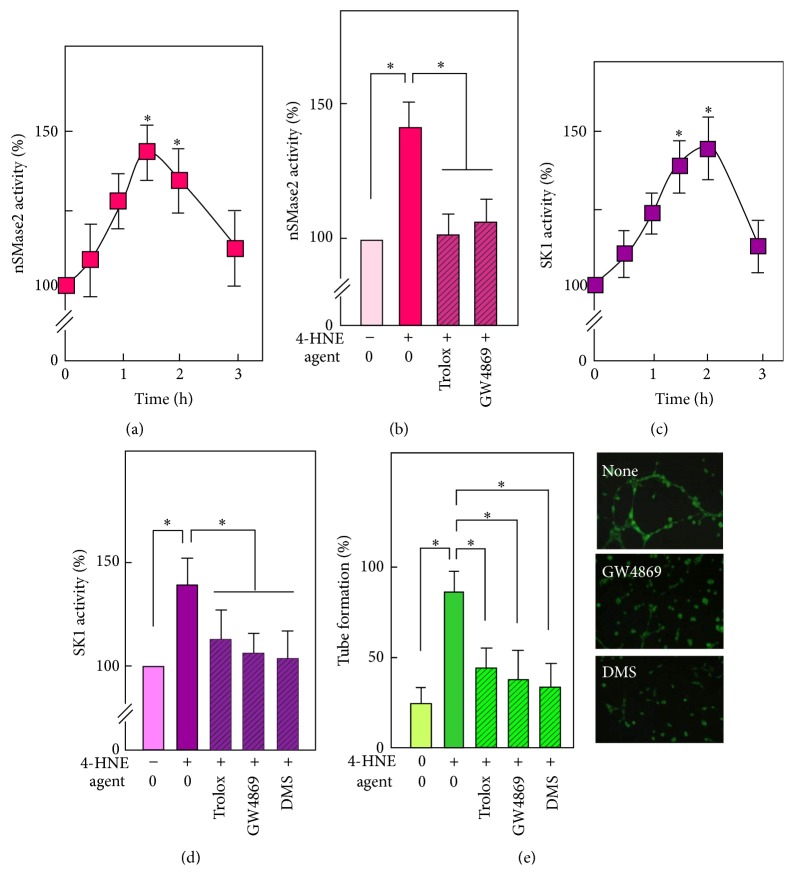
*Implication of nSMase2 and SK1 in tube formation elicited by 4-HNE*. ((a), (b)) Time-course of nSMase2 activation by 4-HNE (0.5 *µ*M) (a) and inhibitory effect of trolox (10 *µ*M) and of the nSMase2 inhibitor GW4869 (5 *µ*M) on nSMase2 activation induced by 4-HNE (b) after 90 min incubation. (c), (d) Time-course of SK1 activation by 4-HNE (0.5 *µ*M) (c) and effect of trolox, GW4869 and of the SK1 inhibitor DMS (1 *µ*M) on SK1 activation by 4-HNE (d), measured after 90 min incubation. (e) Effect of trolox, GW4869, and DMS on capillary tube formation on Matrigel elicited by 4-HNE (0.5 *µ*M). Means ± SEM. ^*∗*^*P* < 0.05; ns: not significant.

As oxLDLs trigger a redox-dependent activation of the neutral sphingomyelinase2/sphingosine kinase-1 pathway (nSMase2/SK1 pathway) which is involved in oxLDL-induced angiogenesis [35, 36], we investigated whether the sphingolipid signaling pathway is implicated in 4-HNE-induced angiogenesis. As shown in Figure 4(a), incubation of HMEC-1 with 4-HNE (0.5 *µ*M) elicits nSMase2 activation, peaking at 90 min, dependent on ROS generation, and inhibited by trolox. Moreover, as expected, nSMase2 activation was inhibited by GW4869, a well-known nSMase2 inhibitor (Figure 4(b)). 4-HNE induced SK1 activation that peaked at 90–120 min (Figure 4(c)). In agreement with the previously reported signaling cascade that coordinates the activation of nSMase2 and SK1 [41], SK1 activation was blocked by trolox that acts upstream from nSMase2, by GW4869 that inhibits nSMase2 but has no direct inhibitory effect on SK1 and by DMS which is a classical inhibitor of SK1 (Figure 4(d)). Interestingly, SK1 inhibition by GW4869 or by DMS was associated with the inhibition of capillary tube formation evoked by 4-HNE (Figures 4(d) and 4(e)), which is consistent with a role of SK1 in S1P generation, in agreement with oxLDL-induced angiogenesis [35, 36].

### 3.5. 4-HNE-Induced Tube Formation Is Blocked by Hydralazine (Hdz) and Bisvanillyl-Hydralazone (BVH)

**Figure 5:**
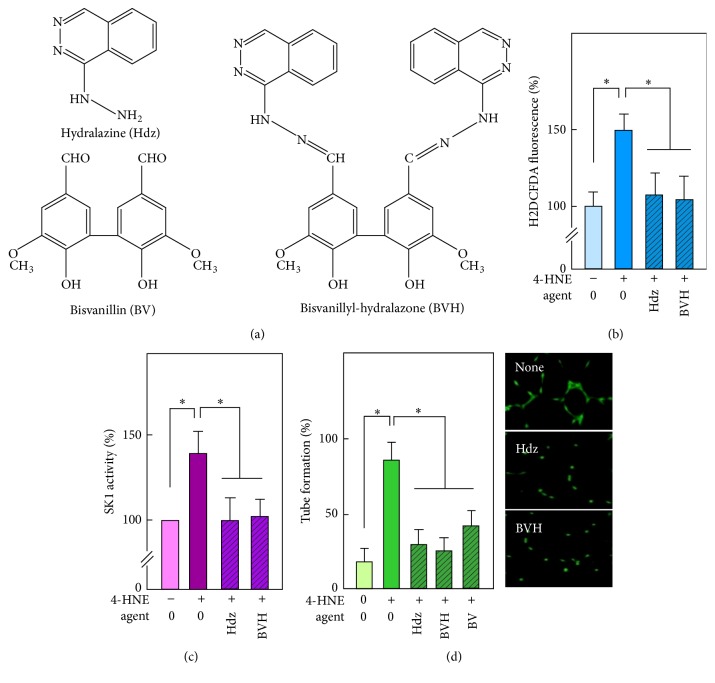
*Effect of hydralazine and BVH on the angiogenic signaling of 4-HNE*. (a) Chemical structures of hydralazine (Hdz), bisvanillin (BV), and bisvanillyl-hydralazone (BVH). (b) Effect of Hdz and BVH (10 *µ*M each), on ROS generated by HMEC-1 after 30 min of contact with 4-HNE (0.5 *µ*M). (c) Effect of Hdz and BVH (10 *µ*M each), on SK1 activation by 4-HNE, measured after 90 min incubation. (d) Effect of Hdz, BVH, and BV (10 *µ*M each) on tube formation elicited by 4-HNE (0.5 *µ*M). These data are means ± SEM of 4 separate experiments. ^*∗*^*P* < 0.05; ns: not significant.

Hydralazine (Hdz) is used for medical purposes as an antihypertensive drug and in combination with isosorbide dinitrate (BiDil) for the treatment of heart failure [42]. Its antiatherogenic effect has been evaluated in several hypercholesterolemic mice models [43–46]. We recently synthesized a new hydralazine derivative, the bisvanillyl-hydralazone (BVH) (Figure 5(a)), which associates antioxidant (bisvanillin) and carbonyl scavenger (hydralazine) activities and prevents both the carbonyl stress and fatty streaks formation in apoE−/− mice [39]. This led us to evaluate whether these carbonyl scavengers may prevent the angiogenic response triggered by 4-HNE in our experimental model system. Both Hdz and BVH inhibited the 4-HNE-induced ROS rise, SK1 activation, and the tube formation by HMEC-1 (Figures 5(b)–5(d)). These data suggest that Hdz may prevent the oxidative stress triggered by 4-HNE and the angiogenic response of endothelial cells.

## 4. Discussion

The data reported in this manuscript show that a low concentration of 4-HNE may stimulate the formation of capillary tubes by HMEC-1 on Matrigel. The angiogenic effect of 4-HNE is mediated through a signaling pathway involving ROS generation and the subsequent activation of the sphingolipid pathway (nSMase2/SK1/S1P). These effects of 4-HNE can be blocked by antioxidants and by inhibitors of the nSMase2/SK1 pathway and are prevented by carbonyl scavengers such as Hdz and BVH.

The highly reactive 4-HNE is generated during lipid peroxidation of n-6 polyunsaturated fatty acids (PUFAs) under various physio(patho)logical conditions [23]. In advanced atherosclerotic lesions, 4-HNE-adducts accumulate in atheromatous areas [26, 27]. Oxidized lipids present in these areas [21] induce a sustained ER stress in vascular cells [27, 46], alter autophagy and efferocytosis, and reduce the mobility of lipid-laden macrophages that are trapped in the lesion [14]. In these areas, neovascularization developed by sprouting angiogenesis from adventitial vasa vasorum invades progressively the atherosclerotic area and takes part in the progression of lesions and complications observed in unstable plaques, such as hemorrhages and rupture [4–7, 47]. Neovascularization may be induced by classical angiogenic pathways, such as hypoxia/HIF/VEGF in the hypoxic thickened intima, and by other atherosclerotic factors, such as inflammation, oxidative stress, and oxidized lipids [36]. RCC-adducts, including 4-HNE-adducts, are highly concentrated in the necrotic lipidic center of the plaque, where lipids are not cleared and autooxidize [27, 48].

At the periphery of the lesions, the density of 4-HNE-adducts is lower, but the fluorescent detection shows a faint staining for 4-HNE-adducts, particularly around CD31 positive cells that form tubular capillary structures (Figure 1). This is consistent with experiments on cell culture showing the angiogenic effect of low 4-HNE concentrations. Similarly, immunohistochemical studies of human aorta revealed the presence of 4-HNE-adducts at low concentration in early atherosclerotic lesions [26], while neovascularization is present at early stages of coronary artery disease and is associated with epicardial endothelial dysfunction [49]. Moreover, in experimental hypercholesterolemia in pigs, coronary neovascularization occurs very early in atherogenesis, prior to endothelial dysfunction [8, 9]. This suggests that the local hemodynamic stress associated with hypercholesterolemia may induce neovascularization, before intimal thickening and local hypoxia, in atherosclerotic prone areas, where inflammation and oxidative stress initiate lipid peroxidation [14, 26, 50]. It is thought that lipid peroxidation occurring in vivo in early atherosclerotic lesions is a slow process that generates low levels of 4-HNE. This is consistent with the angiogenic effect of very low 4-HNE concentration in our HMEC-1/Matrigel system. Interestingly, similar concentration of 4-HNE (0.1 to 1 *µ*M) promotes VEGF expression and secretion in retinal pigment epithelial cells that induce a paracrine angiogenic response [37]. However, under our standard experimental conditions (cell culture in normoxia), HMEC-1 do not release VEGF (or only very low level) and the angiogenic effect cannot be attributed to a VEGF-mediated autocrine angiogenic response. This led us to investigate another angiogenic mechanism by analogy with that involved in oxLDL-induced angiogenesis by HMEC-1 [35]. Low concentrations of oxLDLs trigger capillary tube formation by endothelial cells on Matrigel and angiogenesis in vivo in the Matrigel plug assay [31, 35]. This angiogenic effect of oxLDLs is mediated, at least in part, through LOX-1 and NADPH oxidase activation [31, 35], but our data show that the angiogenic effect of free 4-HNE does not require LOX-1. This is consistent with the specificity of LOX-1 for modified lipoproteins and 4-HNE-modified proteins [51]. Thus, 4-HNE can react with cellular proteins or peptides either at the plasma membrane or inside the cell, as previously reported [24, 52–54]. However, in the reported experiments, the 4-HNE-induced tube formation is not inhibited by anti-LOX-1 blocking Ab, suggesting that the binding of 4-HNE-modified proteins with LOX-1 is not involved in the angiogenic response to 4-HNE. In our model, ROS induced by 4-HNE are generated by a NADPH oxidase, as suggested by the inhibitory effect of DPI and Vas2870 and in agreement with the 4-HNE-induced activation of NADPH oxidase, which is mediated through lipoxygenase activation in macrophages [55].

ROS generated upon 4-HNE stimulation act as an intracellular signaling that activates the nSMase2/SK1 pathway. This role of ROS in nSMase2 activation is consistent with the redox-dependent activation of nSMase2 induced by TNF-alpha [56], daunorubicin [57], H2O2 [41, 58], and oxLDLs [35]. These data are consistent with the inhibitory effect of trolox and of NADPH oxidase inhibitors, DPI and Vas2870 that concomitantly block the intracellular ROS rise, and the activation of nSMase2 and SK1, which also inhibit the activation of the nSMase2/SK1 pathway by oxLDLs [35]. Interestingly, nSMase2 inhibition, either by antioxidants and NADPH oxidase inhibitors or by the specific nSMase2 inhibitor GW4869, inhibits in turn SK1 and angiogenesis. This suggests that nSMase2 plays a crucial role in 4-HNE-induced angiogenesis, as also supported by the angiogenic effect of low C6 ceramide concentration on HMEC-1 grown on Matrigel [36]. Moreover, these data show that SK1 activation depends on nSMase2, since SK1 activation by 4-HNE is blocked when nSMase2 is inhibited, supporting a coordinated signaling cascade as previously reported [41]. Interestingly, although the starting point of angiogenic signaling triggered by oxLDLs and free 4-HNE is not similar (dependent versus independent from LOX-1), these atherogenic compounds trigger an intracellular signaling that induces ROS generation and activation of the nSMase2/SK1 pathway. This is consistent with reports showing that cellular stresses inducing ROS generation activate the sphingolipid pathway [41], which is involved in physiological and pathological vascular biology by regulating endothelial integrity, migration and proliferation, angiogenesis, vascular tone, and leukocyte recruitment [59–62].

Finally, the angiogenic effect of 4-HNE depends on SK1 activation, since its inhibition blocks tube formation, in agreement with the angiogenic effect of S1P [63–65] and with oxLDL-induced angiogenesis [35]. Under the culture conditions used here, 4-HNE did not elicit any significant expression of VEGF, like that observed with oxLDLs. However crosstalks between the SK1/S1P and the VEGF/VEGFR pathways have been reported in the angiogenic effect of S1P and VEGF [66–68] and oxLDLs [35].

Another aim of this study was to investigate the antiangiogenic properties of hydralazine (Hdz), an antihypertensive drug with carbonyl scavenger activity, and of its derivative bisvanillyl-hydralazone (BVH), in which hydralazine is covalently bound to bisvanillin, a phenolic antioxidant. These compounds exhibit a potent antiatherogenic effect in the apoE−/− murine model of atherosclerosis [30, 39]. We report here that both Hdz and BVH prevent the angiogenic effect of 4-HNE. Hdz is a potent carbonyl scavenger that reacts rapidly with 4-HNE and prevents the formation of 4-HNE-protein adducts [30]. BVH, which is constituted by two molecules of Hdz associated with the antioxidant BV, is able to scavenge 4-HNE and block intracellular ROS generated by cells [39]. In our experimental model system, both Hdz and BVH prevent almost completely the angiogenic effect of 4-HNE.

In conclusion, our data emphasize the role of 4-HNE in the formation of tubes evoked by oxidized LDL and suggest that, in vivo, these oxidized lipids may contribute to the neovascularization of atherosclerotic lesions, particularly at the periphery of the plaque where their concentration is lower. In contrast, higher 4-HNE concentration in the lipid core could contribute to the apoptosis of neovessels, thereby promoting intraplaque hemorrhage and plaque rupture. In this context, it could be of interest to evaluate in vivo the ability of pharmacological carbonyl scavengers to prevent the formation of neovessels together with the reduction of atherosclerosis progression.

## Abbreviations

BV: Bisvanillin
BVH: Bisvanillyl-hydralazone
DMS: Dimethyl sphingosine
FBS: Fetal bovine serum
Hdz: Hydralazine
HMEC-1: Human microvascular endothelial cell
4-HNE: 4-Hydroxynonenal
oxLDL: Oxidized LDL
nSMase2: Neutral type 2-sphingomyelinase
RCC: Reactive carbonyl compound
SK1: Sphingosine kinase-1
S1P: Sphingosine 1-phosphate
VEGF: Vascular endothelial growth factor.
